# Characterization of prenatal healthcare for implementation of congenital toxoplasmosis surveillance program: cross-sectional study

**DOI:** 10.1590/1516-3180.2019.0512.R2.10062020

**Published:** 2020-10-26

**Authors:** Aline do Nascimento Benitez, Jancarlo Ferreira Gomes, Milenne Ura Seixas Santos Dias, Italmar Teodorico Navarro, Regina Mitsuka-Breganó, Katia Denise Saraiva Bresciani

**Affiliations:** I MSc, PhD. Veterinarian, Specialist in Zoonoses and Public Health and Postdoctoral Student, Postgraduate Program on Child and Adolescent Health, School of Medical Sciences, Universidade Estadual de Campinas (UNICAMP), Campinas (SP), Brazil.; II MSc, PhD. Parasitologist, Specialist in Public Health and Professor, Postgraduate Program on Child and Adolescent Health, School of Medical Sciences, Universidade Estadual de Campinas (UNICAMP), Campinas (SP), Brazil.; III RN. Nurse, Specialist in Occupational Health and Regional Director, Group XI (GVE), “Prof Alexandre Vranjac” Epidemiological Surveillance Center, State Health Department of São Paulo, Araçatuba (SP), Brazil.; IV MSc, PhD. Parasitologist, Specialist in Zoonoses and Public Health and Professor, Department of Preventive Veterinary Medicine, Universidade Estadual de Londrina (UEL), Londrina (PR), Brazil.; V MSc, PhD. Parasitologist, Specialist in Zoonoses and Public Health and Professor, Department of Preventive Veterinary Medicine, Universidade Estadual de Londrina (UEL), Londrina (PR), Brazil.; VI MSc, PhD. Parasitologist, Specialist in Zoonoses and Public Health and Professor, Department of Animal Production and Health, School of Veterinary Medicine, Universidade Estadual Paulista (UNESP), Araçatuba (SP), Brazil.

**Keywords:** Toxoplasma, Public health, Zoonoses, Protozoan infections, Prevention and control [subheading], Pregnancy, Preventive measures, Health policies, Gestation

## Abstract

**BACKGROUND:**

Prenatal toxoplasmosis remains a neglected disease worldwide and few government programs focusing on its prevention are available. Success in these programs has been extensively reported in the literature, yet the strategies used for their implementation, as a model for such actions in different communities, have not been described.

**OBJECTIVE:**

To describe the aspects of prenatal care strategies in 13 municipalities within the regional healthcare unit of Araçatuba, in the northwestern region of the state of São Paulo in 2017, focusing on congenital toxoplasmosis.

**DESIGN AND SETTING:**

Descriptive study on prenatal healthcare within the Brazilian National Health System, in 13 participating municipalities.

**METHODS:**

Data on serological tests, referral clinics, notifications, healthcare strategies, health education and drugs for infected children were requested through a questionnaire that was sent via e-mail to people responsible for healthcare services in these municipalities.

**RESULTS:**

Major differences relating to diagnoses, reference outpatient clinics, notifications, health education and healthcare and drugs for infected children were reported among the prenatal strategies of these 13 municipal healthcare services.

**CONCLUSION:**

The lack of standardized prenatal strategy in the study area may compromise the prevention of infection. However, our identification of each aspect of prenatal care corroborates the need to implement a healthcare surveillance program relating to congenital toxoplasmosis.

## INTRODUCTION

The transmission routes for toxoplasmosis include consumption of raw foods, undercooked meats and untreated water; contact with soil contaminated with the evolutionary infective forms of the protozoon *Toxoplasma gondii* (*T. gondii*); and transplacental infection.^1^ Differences in pregnant women's food intake and cultural habits around the world are responsible for variations in the prevalence of congenital toxoplasmosis among countries,^2^ and between communities within the same nation.^3^ Preventive measures are common to all populations, since they concentrate on health education and early detection of infection during pregnancy.^4^ However, the public health strategies for prevention of this parasitosis need to be appropriate for each situation.

*T. gondii* infection is a major problem within gestational health because of the possibility of irreversibly damaging the fetus.^1^ The main types of damage reported have included chorioretinitis, deafness, intracranial calcification, microcephaly, hydrocephalus, seizures and intellectual disability. In addition, occurrences of spontaneous abortions, neonatal death and neurodevelopmental disorders have been recorded.^5^

Researchers from the World Health Organization (WHO) reported that in 2010, despite neglect due to little attention given to the prevalence, prevention and treatment of toxoplasmosis, 10.3 million cases of this disease were recorded worldwide, with 825,000 Disability-Adjusted Life Years (DALYs), one of the highest among foodborne parasitic diseases.^2^

The Brazilian strains of *T. gondii* are highly damaging to the eye tissue, in comparison with strains in other countries.^6^ However, reduction of the incidence of congenital infection, through early identification of maternal infection and adoption of strategic therapy for pregnant women and newborns up to one year of age, has been observed.^7^ In Switzerland, three decades of serological monitoring of pregnant women led to an 85% reduction in the incidence of congenital toxoplasmosis.^8^ Similar results have been found in other countries such as France^9^ and Austria.^10^

Technical and scientific standards for promotion, protection and recovery of health have been developed within the Brazilian National Health System (Sistema Único de Saúde, SUS). SUS has the mission of promoting public health actions to ensure conditions of physical, mental and social wellbeing for the entire population. This is implemented by the Brazilian Ministry of Health and by the state and municipal health departments, through socioeconomic policies aimed at diminishing the risks of diseases, in a hierarchical flow^11^

Hence, Brazilian states have adopted policies consisting of preventive strategies against congenital toxoplasmosis, with protocols for diagnosis, attendance and treatment among mothers and children, at public healthcare services.^12^ These strategies have been established and validated in the state of Paraná and are available online under the name *“Mãe Paranaense”* (“Paraná-born Mothers”).

In the northwestern part of the state of São Paulo, professionals responsible for prenatal healthcare among pregnant women are instructed to follow the primer of the Brazilian Ministry of Health. This primer provides guidelines for three serological investigations of anti-*T. gondii* antibodies among pregnant women living in regions of high endemicity However, the lack of studies to certify situations of high endemicity of congenital toxoplasmosis may corroborate the perception that negligence in disease notification has been occurring. Although gestational and congenital toxoplasmosis were included through Ordinance no. 204, of February 17, 2016, among compulsory-notification diseases for which weekly reports and standardized guidelines and recommendations are required, some states have not registered any cases yet.

Hence, to improve the detection of gestational toxoplasmosis cases and to reduce occurrences of congenital toxoplasmosis, researchers and postgraduate students at a public university in the state of São Paulo proposed the implementation of a healthcare surveillance program for congenital toxoplasmosis in the northwestern region of the state of São Paulo, to be established in six steps in accordance with the *Mãe Paranaense* guidelines.^13^^,^^14^

## OBJECTIVE

Through this study, we aimed to describe the aspects of prenatal care strategies in 13 municipalities within the regional healthcare unit of Araçatuba, in the northwestern region of the state of São Paulo in 2017. We focused on congenital toxoplasmosis and ascertained the possibility of implementing a surveillance program for congenital toxoplasmosis in this regional healthcare unit.

## METHODS

In the state of São Paulo, according to Decree DOE no. 51,433, of December 28, 2006, healthcare is distributed into 17 Regional Health Departments (Departamentos Regionais de Saúde, DRS), which are responsible for coordinating the activities of the State Health Department with the municipalities and civil society, in order to promote better quality of life for the population of its coverage area. The region of Araçatuba is included in DRS II, which is composed of 40 municipalities covering about 724,570 inhabitants. These municipalities are grouped into three regional management board groups, namely: “Central,” “dos Lagos,” and “dos Consórcios”.

All municipal healthcare managers present at the board meetings of the Regional Inter-Agency Committee (Comissão Intergestora Regional, CIR) were invited to participate in the project, which had the aim of improving the healthcare provided for pregnant women with congenital toxoplasmosis, through academic-scientific partnerships between epidemiological surveillance bodies and municipal health departments.

Among the municipal managers who agreed to participate, some information was requested through a questionnaire that was sent out via e-mail to each municipal health department. The data recorded were grouped in terms of the following general characteristics: infrastructure available in the municipalities for prenatal care and for making the serological diagnosis; the time at which trimestral screening was performed and the parameters adopted for this; and the confirmatory test and the place and circumstances used for notification of the infection (Table 1); and also according to the drug intervention (Table 2).

**Table 1:** Description of general characteristics of the healthcare provided towards preventing congenital toxoplasmosis, as reported by the 13 municipal health departments of the Regional Health Department II, in 2017

Parameter	Municipality													Reference*
	1	2	3	4	5	6	7	8	9	10	11	12	13	
General characteristics														
Number of PHUs for prenatal care	18	2	10	2	1	1	1	1	1	1	9	1	1	§
Number of pregnant woman receiving healthcare	1890	46	1100	89	*	34	20	200	21	30	358	*	42	§
Presence of ESF	Yes	Yes	Yes	Yes	Yes	Yes	Yes	No	Yes	Yes	Yes	Yes	Yes	Yes
Veterinarians in the NASF	No	No	No	No	No	No	No	No	No	No	No	No	No	Yes
Personal educational activities	Yes	No	No	No	Yes	Yes	No	No	No	Yes	No	No	Yes	Yes
Monthly prenatal appointments	6	8	6	9	6-7	12	10-12	8-10	6-14	8	9	14	9-12	§
Minimum number of US per pregnancy	2	3	2	*	2	4	12	3	3	3	2	7	5	4
Medical team available at prenatal visits	Yes	Yes	Yes	Yes	Yes	Yes	Yes	Yes	Yes	Yes	Yes	Yes	Yes	Gynecology
	No	No	No	No	No	No	No	No	No	No	No	No	No	Obstetrics
	No	No	No	No	No	No	No	No	No	No	No	No	No	Infectiology
	No	No	No	No	No	No	No	No	No	No	No	No	No	Pediatrics
Infrastructure	Yes	No	Yes	Yes	Yes	Yes	No	Yes	Yes	Yes	Yes	Yes	Yes	PHU
**Diagnostics**														
Number of laboratories	3	1	6	1	0^a^	2	*	1	1^a^	2	2	1	1	§
Blood sample collection at PHU	No	Yes	No	Yes	No	Yes	No	No	Yes	Yes	No	Yes	No	§
**Trimestral screening**														
Was screening done every trimester?	No	No	No	No	No	No	No	No	No	No	No	No	No	Yes
Trimesters	1, 2	1, 2	1, 3	1, 3	1, 3	1, 2	1, 2	1	1	1, 3	1, 2	1	3	1, 2, 3
Serological methods	Yes	No	Yes	No	Yes	No	No	No	Yes	No	Yes	No	No	CMIA
	Yes	No	Yes	No	No	Yes	No	No	No	No	No	No	No	MEIA
	Yes	No	Yes	No	No	No	No	No	No	No	No	No	No	ELFA
Quantified antibody titers	Yes	Yes	No	No	Yes	Yes	No	Yes	Yes	Yes	Yes	Yes	Yes	Available
**Confirmatory test**														
Parameter used to confirm infection	No	Yes	Yes	Yes	No	No	-	Yes	No	Yes	Yes	Yes	Yes	IgG avidity test
	No	No	No	No	No	No	No	No	No	No	No	No	No	IgG+ IgM- until 16^th^ GW
Local laboratory	Yes	No	Yes	Yes	No	Yes	-	-	No	Yes	Yes	Yes	Yes	§
Screening sample used for confirmation	Yes	Yes	Yes	No	-	-	-	No	-	Yes	No	No	Yes	Yes
Time taken to obtain the result		2	7	7	-	15	-	10	-	20	5	7	20	§
Serological tests on NB	Yes	Yes	Yes	No	Yes	Yes	Yes	Yes	Yes	Yes	Yes	No	Yes	In gestational infection
**Notification**														
Notification at PHU	Yes	Yes	Yes	Yes	Yes	Yes	Yes	Yes	Yes	Yes	Yes	Yes	Yes	At PHU
Circumstances for notification	No	No	No	No	No	No	No	No	No	No	No	No	No	IgG and IgM Seroconversion Until the 16^th^ GW:
	No	No	No	No	No	Yes	No	No	No	No	No	No	Yes	IgG+ IgM+ and Low IgG avidity.
	No	No	No	No	No	No	No	No	No	No	No	No	No	After 16^th^ GW: IgG+ IgM+

* Reference according to the implementation described by Mitsuka-Breganó, Lopes-Mori and Navarro, 2010.^14^

PHU = primary healthcare unit; ESF = Family Health Strategy (from the Portuguese “Estratégia Saúde da Família”); NASF = Family Health Support Center (from the Portuguese Núcleo de Apoio à Saúde da Família); NB = newborn; US = ultrasound; GW = gestational week; CMIA = chemiluminescent microparticle immunoassay; MEIA = microparticle enzyme immunoassay; ELFA = enzyme-linked fluorescent assay.

**Table 2:** Description of drug interventions within the healthcare provided to pregnant women for preventing congenital toxoplasmosis, as reported by the 13 municipal health departments of the Regional Health Department II, in 2017

Information	Municipality													Reference*
	1	2	3	4	5	6	7	8	9	10	11	12	13	
Circumstance for prescription	No	No	No	No	No	No	No	No	No	No	No	No	No	IgG e IgM seroconversion.
	No	No	No	No	No	No	No	No	No	No	No	No	No	IgM +
Medicine	Yes	Yes	Yes	Yes	Yes	Yes	Yes	Yes	-	Yes	Yes	Yes	Yes	Spiramycin
	No	Yes	No	No	No	Yes	No	No	No	No	No	No	No	Folinic Acid
	No	Yes	No	No	No	Yes	No	No	No	No	No	No	No	Sulfadiazine
	No	No	No	No	No	Yes	No	No	No	No	No	No	No	Pyrimethamine
Drug intervention according to GW	No	No	Yes	No	No	Yes	No	No	No	No	No	No	Yes	Yes
Immediate availability of IgG avidity test	Yes	Yes	No	No	Yes	No	Yes	No	No	Yes	Yes	Yes	No	Yes
Days elapsed from blood collection to administration of medicine	7	15	30	7	60	15	7-10	15-30	30-40	20-30	7	15	-	§

* Reference according to the implementation described by Mitsuka-Breganó, Lopes-Mori and Navarro, 2010.^14^

§ Information not available regarding a reference; GW = gestational week; IgG = immunoglobulin G; IgM = immunoglobulin M.

Information from each municipality was described individually and differences between strategies were recorded.

The manual “Gestational and congenital toxoplasmosis: health surveillance, diagnosis, treatment and conduct”, which was created in the city of Londrina, Paraná, in 2006, was used as reference material. This material was then implemented in the state of Paraná as part of the program “Paraná-born Mothers – Books for healthcare provided for combating prenatal toxoplasmosis”.^15^

The study “Implementation of the healthcare surveillance program for congenital toxoplasmosis in the northwestern region of the state of São Paulo” was approved by the local research ethics committee on April 27, 2018 (opinion report no. 2,625,140).

## RESULTS

Out of the 40 municipalities that form the regional healthcare unit of Araçatuba, 13 agreed to participate in this project. In 12/13 municipalities (92.30%) that were surveyed, prenatal care is carried out in outpatient clinics at primary healthcare units. Between 17 and 110 prenatal medical appointments take place at each unit per month. In those with more than 45 prenatal appointments per month, the services of the teams of the Family Health Strategy (Estratégia Saúde da Família, ESF) or the Family Health Support Center (Núcleo de Apoio à Saúde da Família, NASF) are available in order to develop complementary actions to promote pregnant women's health. However, in 1/13 municipalities (7.7%), these teams are unavailable. In that municipality, healthcare for all the 200 pregnant women is concentrated at an institution that specializes in promoting women's healthcare.

Educational activities relating to toxoplasmosis for pregnant women are carried out in 8/13 municipalities (61.53%) as stated in the strategies of the *“Mãe Paranaense”* guidelines. In five of the municipalities, no such action is carried out. In the other three municipalities, preventive information on infection is given collectively to groups of pregnant women.

Blood samples are collected from the pregnant women attended in all the municipalities, either directly at laboratories or at the primary healthcare unit, for forwarding to the laboratory. These laboratories for investigating anti-*T. gondii* immunoglobulin (Ig)G and IgM antibodies are either in the municipality or in a nearby town. In 1/13 municipalities, blood from pregnant women is collected at a laboratory in a nearby municipality.

Surveillance for gestational toxoplasmosis is performed once in 4/13 municipalities (30.76%), and twice in the remainder of the localities. In 12/13 municipalities (92.30%), the healthcare strategy involves screening in the first semester.

The serological methods of chemiluminescent microparticle immunoassay (CMIA), microparticle enzyme immunoassay (MEIA) or enzyme-linked fluorescent assay (ELFA), with quantitative presentation of antibodies, are used in 7/13 municipalities (53.85%). In the others, i.e. 6/13 municipalities (46.15%), this information is not obtained.

The test for avidity of IgG is not performed in 4/13 municipalities (30.76%). IgM-positive blood samples are forwarded for performing the avidity test in neighboring towns. This strategy is feasible for most of the municipalities because the results become available within two days. However, in two municipalities, the reports may take between 10 to 15 days, even though there is a laboratory in each of these municipalities. In 3/10 municipalities (30.00%), a new blood sample is collected for the avidity test.

The number of prenatal appointments per pregnant women ranged between six and 14, with completion of 2 to 12 ultrasound scans per patient. Only in one municipality is this diagnostic imaging test not performed.

Investigation of the presence of anti-*T gondii* antibodies in infants, as part of the daily work routine, is not recommended in 12/13 municipalities (92.30%). In one municipality, this test is not performed even when there is a confirmed case of gestational infection.

Classification of pregnant women at risk of congenital toxoplasmosis, based on the serological results, is not carried out in one municipality; and in two there was no answer regarding this question. Concerning the criteria used to make the notification, the answer was “after confirming the infection” in 9/13 municipalities, while there was no response from one and the answer from two was incomplete, since it was stated that notification was only performed in cases in which the pregnancy was not more than 16 weeks.

Therapeutic intervention was inadequate in all the municipalities. According to the responses, it was implemented “afer medical prescription,” “afer serological result” or “as soon as possible” in 8/13 municipalities. In the others, it was done after waiting for the result from the avidity test. Spiramycin was reported to be the active substance of choice by 12/13 municipalities.

In only 2/13 municipalities, the pregnant woman is reevaluated, and the choice of therapy is guided according to the gestational week (GW). Although this conduct is in accordance with the *“Mãe Paranaense”* guidelines, there is no immediate availability of spiramycin for pregnant women in 6/13 municipalities. The time between blood collection and the beginning of the treatment was described as between 7 and 60 days.

Detailed information about each municipality is available in Tables 1 and 2.

## DISCUSSION

The strategies to prevent gestational toxoplasmosis during the prenatal period in the 13 municipalities surveyed in this region are not standardized. Overall, aspects such as the diagnosis, reference outpatient clinics, notification, health education activities and the medicines and healthcare provided for infected children differ. These differences may hinder earlier detection of gestational infection by *T. gondii.*

Among the actions adopted by these 13 municipalities in the northwestern region of the state of São Paulo in order to avoid infection by *T. gondii* in pregnant women, we identified particular matters that differed from what is recommended in the reference material used in our study, i.e. “Gestational and congenital toxoplasmosis: health surveillance, diagnosis, treatment and conduct.” These differences have the potential to undermine the implementation of the prevention program for congenital toxoplasmosis in this region.

These weaknesses allow us to suggest interventions to minimize possible failures when undertaking actions among pregnant women, individually, per municipality. The overall aspects of the preventive healthcare actions against congenital toxoplasmosis adopted by municipal governments for prenatal care through SUS can be individually searched, in order to conform with the structure provided and adapt the strategies to the available resources.^16^

Analyses on the general characteristics of the preventive healthcare actions against congenital toxoplasmosis (Table 1 ) showed that primary healthcare units or healthcare units specializing in women's health were the places for completion of prenatal care in the municipalities surveyed. This information was provided by the respondent healthcare managers of the 13 municipalities investigated, through the questionnaires.

Through the ESF and NASF teams, more professionals are available to promote healthcare among pregnant women. These teams are available in municipalities where more than 45 pregnant women are attended per month. They were created in Brazil to reorganize primary healthcare, with interprofessional healthcare teams working in an integrated manner. Trough this, the capacity for analysis and intervention concerning health problems and needs within the area of coverage is increased, in clinical, sanitary and environmental terms.^17^ Thus, if toxoplasmosis is addressed by different professionals, knowledge about the disease is corroborated, which may increase the chances that it will be recognized in pregnant women in recognizing and that the risks of infection can be avoided.

The presence of members of the community in these teams, such as community health agents (agentes comunitários de saúde, ACS), facilitates creation of bonds with the population and interactions with the team of healthcare professionals.^18^ Thus, indirectly, this assists in health education and in actively seeking out pregnant women to ensure that they receive complete prenatal follow-up. Incorporation of veterinarians and graduates in epidemiology, public health, zoonoses and food inspection, among others, in NASF teams is highly recommended, in order to add quality to the dissemination of knowledge to the population. Successful work by veterinarians has been reported in relation both to prevention of zoonoses^19^^,^^20^ and to responsible conduct towards companion animals.^19^ It is paramount that women understand the *T. gondii* cycle and its transmission routes, in order to avoid infection during pregnancy.^21^

In the states of Rio de Janeiro and Paraná, lack of knowledge about toxoplasmosis was observed among, respectively, 232/405 (57.28%) and 177/330 (53.63%) pregnant women attended through the municipal public healthcare network.^22^ In a city in the northwestern region of the state of São Paulo, an investigation on the degree of knowledge about toxoplasmosis and leishmaniasis among 123 residents showed that 29/123 (57.0%) of the respondents did not recognize the term “zoonosis,” 68/123 (55.3%) did not know how to prevent toxoplasmosis and, although 119/123 (96.7%) stated that they knew what leishmaniasis was, dogs were deemed to be transmitters of infection through their feces, urine, bites or licking, or believed that transmission occurred through animal blood, mosquito bite or the mosquitos' contaminated feces mosquitos.^23^ This result confirmed that there is a need for dissemination of knowledge about the *T. gondii* cycle, concerning the disease transmission route and symptoms and the damage caused to fetuses, in order to intensify the strategy of prevention of gestational infection.

Health education for pregnant women should be conducted on a one-to-one basis, to achieve better results. This was seen among pregnant women in a municipality in Paraná, Brazil, who reported that they gained knowledge of toxoplasmosis through lectures (120/153; 78.4%), group reading (24/153; 15.7%) and pamphlets (15/153; 9.8%). Better knowledge of preventive strategies against congenital and gestational toxoplasmosis has been correlated with health professionals' participation in an integrated network.^21^

Early diagnosis is crucial for preventing congenital infection. Some aspects of how laboratory tests are organized, such as daily test production capacity and the sample collection site, can reduce the time taken to detect acute infection.

There is a lack of specific guidance from the Brazilian Ministry of Health regarding the place at which blood collection should be performed, for investigating the presence of anti-*T. gondii* antibodies. Nonetheless, shortening the time taken to implement therapeutic intervention after pregnant women become infected is crucial for reducing the damage to fetuses.^24^ In the state of Minas Gerais, Brazil, all blood samples from pregnant women are collected on filter paper and are sent to the reference laboratory within 24 hours, using a specialist postal service for transporting this material. The result is made available online, with consequent release of the therapeutic drug through SUS, if necessary. This highly organized system in Minas Gerais shows that, regardless of the place at which blood samples are collected, efforts should be concentrated on implementation of a flow process for transportation of samples, so as to optimize the time that elapses between blood collection and drug interaction.

Diagnostic screening for gestational toxoplasmosis, which is recommended at the beginning of each gestational trimester, was the matter of greatest divergence from the guidelines. This was not incorporated within the healthcare strategy of any of the municipalities in our research.

Infections in the last trimester cause mental disorders and hearing impairments that are often manifested after birth.^25^^,^^26^ This late diagnosis may be related to negligent evaluation in the third trimester. However, since the rate of vertical transmission of *T. gondii* increases with increasing gestational age, trimestral serological evaluation, at least, is essential within surveillance programs for congenital toxoplasmosis.^9^ Clinical patterns of asymptomatic acute infection, which may occur at any stage of pregnancy, can only be demonstrated through serological monitoring. Detection of these cases increases the chances that the baby will grow without after-effects, since it triggers a flow of actions for early and continuing treatment during pregnancy and after birth. Moreover, the costs of treatment of the disease due to infection at the end of pregnancy may be higher than the costs of serological monitoring within prenatal care. Such situations may have an impact on a country's economy. This was seen in Austria, where the government was able to save 258 million euros over a 17-year period through bimonthly serological evaluations on pregnant women. These evaluations reduced the extent of damage caused by congenital toxoplasmosis.^10^

The diagnostic methodology needs to include quantification of antibodies in order to aggregate information that can guide the medical conduct until acute infection has been confirmed through laboratory data. By making this quantitative information available, antibody curves for paired samples over 15-day intervals can be determined. This aids in interpreting the results from IgM-reactive individuals. Another way to confirm infection is to use an IgG avidity test on samples that were positive for IgM in screening tests.^14^

To make this diagnosis, it is essential to define a flowchart for confirming recent infections. IgG avidity tests should be requested within the first 16 weeks of pregnancy, if the pregnant woman is positive for IgG and IgM in the screening test. To achieve this, urgency in temporal dynamics between conducting tests on samples and beginning the treatment for congenital toxoplasmosis is required. Thus, we suggest that in the cases of the two municipalities, in which the reports are received only 20 days after the samples were collected, alternatives need be discussed with the aim of shortening these temporal dynamics. The healthcare managers responsible for prenatal care should advise the laboratory service to perform the avidity confirmatory test on the same sample as used for screening.

In this proposal for a program for preventing toxoplasmosis, efforts need to be made to facilitate drug interventions in urgent cases of gestational toxoplasmosis. To do so, it is paramount to define a strategic flowchart with diagnostic actions that go from taking samples from pregnant women to production of the diagnostic report that is to be given to the doctor responsible for the prenatal care.^13^

Prenatal care in the municipalities surveyed was unsatisfactory. Moreover, there was a need to change the number of ultrasounds performed, in order to achieve bimonthly monitoring, and monthly monitoring in cases in which changes to the fetus are observed. Trough ultrasounds, conditions such as ventriculomegaly, higher hepatosplenic or periventricular density, focal injuries to the brain tissue, calcification, punctate lesions, fetal ascites or greater placental thickness can be ascertained.^27^

In Brazil, compulsory notification for gestational toxoplasmosis has only been established in sentinel units. These units are used in Brazil to improve the notification of diseases without any use of a specific questionnaire for epidemiological investigation. Thus, it might be possible to aim towards a better notification service for acute toxoplasmosis if no sentinel units are available.

Classification of the risk of congenital toxoplasmosis and the criteria for notification need to be intensified. This is especially important after two-thirds of pregnancy has passed. This is the point at which the greatest lack of monitoring has been reported. This lack of monitoring may be due either to lack of prenatal follow-up on the part of pregnant women, or to lack of requests for serological tests at this time, which was reported in several municipalities. The recommendation given in the program is that detection of IgG and IgM-reactive samples with low IgG avidity should be notified until the 16^th^ gestational week, and that seroconversion of IgG and IgM when IgG and IgM are reactive should be notified at any time during prenatal care.^15^

The level or quality of therapeutic intervention reflects the success of newborn recovery and damage reduction.^14^ In 84.6% of the municipalities, the quality of therapeutic intervention was insufficient, especially regarding the adequacy of active substances that were administered after confirmation of acute gestational toxoplasmosis, but also concerning immediate availability of spiramycin and the time between blood collection and the beginning of the treatment.

Use of spiramycin in gestational toxoplasmosis protects the fetus from ocular damage, as observed in a cohort study conducted in Colombia. In that study, ocular toxoplasmosis was found in 1/15 children (6.6%) from pregnant women who used spiramycin, whereas it was found in 5/8 babies (62.5%) from untreated pregnant women, and in two of these cases, involvement of the central nervous system was verified.^28^

In a cohort study conducted in Goiânia (GO), Brazil, congenital infection was detected in 58.33% of newborn babies from pregnant women who received treatment with spiramycin, and in 18.6% of them the damage was severe. On the other hand, among untreated pregnant women, infection was found in 73.04% of their babies, with severe damage to health in 60.7% of them.^4^

In treating gestational toxoplasmosis to avoid congenital toxoplasmosis spiramycin needs to be added. This acts as a parasitostatic agent on the placental barrier. Sulfadiazine and pyrimethamine antiparasitics are added to act on fetal tissues. Folinic acid needs to be administered whenever its antagonist, pyrimethamine, is activated.^15^

Trough the instrument used, we were able to see that actions conflicting with the strategy proposed were occurring in all the participating municipalities at this step of implementation, regarding all aspects evaluated. The healthcare strategies seen in all the municipalities investigated in our study presented at least one aspect that diverged from what is described in the program “Paraná-born Mothers – Books for healthcare provided for combating prenatal toxoplasmosis”.

The profile of preventive healthcare actions, screening and confirmatory diagnoses, prenatal care, healthcare provision for new-borns, notifications and drug interactions can guide the actions for the next stage of implementation of the healthcare surveillance program for congenital toxoplasmosis in the northwestern region of the state of São Paulo.

## CONCLUSION

It needs to be acknowledged that the methodology used in the present study, i.e. interpretation of data collected via email, may have led to some bias. However, to the best of the authors' knowledge, no guidelines on how to start a preventive program for gestational and congenital toxoplasmosis currently exist. Therefore, the simple investigative methodology presented here can be used in other locations as the first stage in implementing a preventive program for combating gestational and congenital toxoplasmosis.

We observed differences in the prenatal strategies among the municipalities surveyed. However, the differences in strategies that were detected confirm that there is a need to implement a healthcare surveillance program for congenital toxoplasmosis.
